# The microbial composition of larval airways from *Drosophila melanogaster* differ between specimens from laboratory and natural habitats

**DOI:** 10.1186/s40793-023-00506-9

**Published:** 2023-06-27

**Authors:** Hanna Angstmann, Stefan Pfeiffer, Susanne Kublik, Birte Ehrhardt, Karin Uliczka, Klaus F. Rabe, Thomas Roeder, Christina Wagner, Michael Schloter, Susanne Krauss-Etschmann

**Affiliations:** 1 Division of Experimental Asthma Research, Early Life Origins of Chronic Lung Disease, Research Center Borstel, German Center for Lung Research (DZL), Airway Research Center North (ARCN), Leibniz Lung Center, Borstel, Germany; 2 ZIEL - Institute for Food and Health, Technical University of Munich, Freising, Germany; 3 Research Unit for Comparative Microbiome Analysis, Helmholtz Zentrum München, Oberschleißheim, Germany; 4 Department of Pneumology, Lungen Clinic, Grosshansdorf, Germany; 5 Department of Medicine, Christian Albrechts University, Germany Member of the German Center for Lung Research, Kiel, Germany; 6 Division of Molecular Physiology, Institute of Zoology, Christian-Albrechts University, Airway Research Center North (ARCN), German Center for Lung Research (DZL), Kiel, Germany; 7 Department of Medicine, Institute for Experimental Medicine, Christian Albrechts University, Kiel, Germany

**Keywords:** *Drosophila melanogaster*, Airway microbiome, Intergenerational transmission, Habitat dependent bacterial genera composition, Immunodeficiency

## Abstract

**Background:**

The fruit fly *Drosophila melanogaster* lives in natural habitats and has also long been used as a model organism in biological research. In this study, we used a molecular barcoding approach to analyse the airways microbiome of larvae of *D. melanogaster*, which were obtained from eggs of flies of the laboratory strain w^1118^ and from immune deficient flies (NF-kB-K), and from wild-caught flies. To assess intergenerational transmission of microbes, all eggs were incubated under the same semi-sterile conditions.

**Results:**

The airway microbiome of larvae from both lab-strains was dominated by the two families *Acetobacteraceae* and *Lactobacillaceae*, while larvae from wild-caught flies were dominated by *Lactobacillaceae, Anaplasmataceae* and *Leuconostocaceae*. Barcodes linked to *Anaplasmataceae* could be further assigned to *Wolbachia sp.*, which is a widespread intracellular pathogen in arthropods. For *Leuconostoceae*, the most abundant reads were assigned to *Weissella sp.* Both *Wolbachia* and *Weissella* affect the development of the insects. Finally, a relative high abundance of *Serratia* sp. was found in larvae from immune deficient relish^−/−^ compared to w^1118^ and wild-caught fly airways.

**Conclusions:**

Our results show for the first time that larvae from *D. melanogaster* harbor an airway microbiome, which is of low complexity and strongly influenced by the environmental conditions and to a lesser extent by the immune status. Furthermore, our data indicate an intergenerational transmission of the microbiome as shaped by the environment.

**Supplementary Information:**

The online version contains supplementary material available at 10.1186/s40793-023-00506-9.

## Background

The airway microbiome is considered an important contributor to the development of chronic respiratory diseases, such as asthma and chronic obstructive pulmonary disease [1–4]. Given its limited accessibility, the respiratory microbiome is however difficult to study in humans. The fruit fly *Drosophila melanogaster* is a recognized model to investigate the molecular underpinnings of chronic airway diseases [5, 6]. However, it is not yet known whether it harbors a respiratory microbiome. The airways of *Drosophila* larvae consist of two tracheal tubes that extend along the horizontal body-axis and ramify into a network of smaller branches [7]. The gas exchange occurs through two openings (spiracles) at the posterior end of larvae [8]. The area of spiracles ranges between 2200 and 2800 µm^2^ [9] which is in principle large enough for bacteria to enter. On the other hand, the spiracular lumen is lined by fine cuticular threads with hair-like extensions that function as air filter and could prevent bacterial immigration [7, 10].

Aside from its presence in nature, *D. melanogaster* is used as model organism in basic and applied research for decades. Our question of interest in this study was whether tracheae of wild flies have a microbiome and, if so, whether it has a different composition from that of flies reared for generations under laboratory conditions. We therefore analysed the tracheal bacterial community structure of larvae descending from flies caught in a natural habitat compared to laboratory strains kept under semi-sterile conditions using a molecular barcoding approach. Eggs from those flies were incubated under identical conditions, which enabled us to analyse the consequences of different parental exposure for the airway microbiome of the next generation. To assess the influence of the immune system on bacterial genera composition, we compared a widely used laboratory wildtype strain to an immunodeficient mutant.

## Methods

### Fly strains

We used the laboratory wildtype line w^1118^ (RRID:BDSC_5905), which was kept for at least 70 generations in our lab and the immune deficient line relish^−/−^ (rel^E38^, RRID:BDSC_9458) lacking a functional version of the transcription factor relish (homologue of mammalian NF-κB precursors p100). About 60 adult wild-living *D. melanogaster* specimens were captured with two commercially available live traps (Trapango®), using fruits as bait, in a domestic kitchen in Bad Oldesloe, Germany (WT-BO, tab. S1). Traps were closed after one day and flies were immediately taken to the lab and directly transferred to a vial with sterile standard medium (supplemented with Methyl 4-hydroxybenzoate and propionic acid to exclude growth of fungi) or egg deposition for 24 h at 25 °C.

### Culture conditions

Laboratory lines were reared on sterile standard cornmeal/molasses/yeast/agar medium at 25 °C and a relative humidity of 50–60% at a 12 h:12 h light:dark cycle.

To obtain time-synchronized larvae for tracheal preparation, each 10 males and females of the parental generation were transferred into a culture tube with sterile standard medium for egg deposition for 24 h at 25 °C. After oviposition adult flies were removed from the vial and eggs were collected.

### Preparation of trachea

To avoid contamination during sample preparation, all instruments were sterilized for 30 min. using UV irradiation under a sterile bench, followed by RNAse away treatment. Subsequently, all instruments were individually wrapped in aluminium foil and sterilized in a sterilizator at 180 °C for 4 h. All liquids were sterile filtered, before use.

To prevent contamination via the skin by the culture medium in which the larvae are grown, the larvae were washed three times (sterile PBS − 70% ethanol - sterile PBS) before being placed in a sterile preparation dish with 150 µl sterile PBS puffer under a sterile hood.

Larvae undergo three developmental stages, which are L1, L2 and L3. We used L3 larvae for microscopic isolation of airways as larvae at earlier stages are more fragile and smaller with a correspondingly higher risk of harming other organs during the preparation of airways. The animals were prepared at a magnification of 25x to 50x under a stereomicroscope. First, the head was completely removed from the larvae using two Dumont forceps. The larvae were then fixed with one forceps, and the dorsal trunk with primary and secondary branches was prepared with another forceps (s. supplemental Video). Remaining body tissue was removed. During the dissection of the respiratory tracts, only those tracheae that were still filled with gas were isolated to exclude any accidental contamination by the sterile preparation buffer that might have occurred during the isolation procedure. Most importantly, the intestine was not damaged during tracheal isolation. When the larvae were opened, only the skin was opened, and the trachea was cut at both ends of the larva and removed as a strand (see video). All other internal organs therefore remained completely untouched. The only contamination could come from haematocytes, which however cannot have an influence on the microbiome as the haemolymph is sterile. After isolation, the tracheae were then washed twice in sterile PBS again to wash away any unintentional external contamination and then transferred to an Eppendorf tube with 150 µl sterile lysis buffer (RA1 buffer, MN NucleoSpin RNA II Kit, Macherey Nagel, Germany) on ice.

Finally, the tracheae from 40 larvae were pooled for one sample per replicate (3 samples for each genotype were treated as true replicates), were homogenized (Pellet pestles, cordless motor; Sigma Aldrich), shock frozen in liquid nitrogen and stored at -80 °C until further processing. Three biological replicates were taken per genotype (see table S1). To prove contamination free preparations negative controls were included in the analysis, including sterile PBS as well as PBS exposed to room air while preparation.

### Microbiome analysis

We used an RNA based pipeline for further analysis as this was less prone to contamination by environmental microbes than a DNA based pipeline (data not shown). Extraction of RNA, cDNA synthesis and amplification of cDNA obtained from 16 S rRNA followed standard protocols.

#### RNA-Isolation and cDNA preparation

Bacterial cells were disrupted using NucleoSpin^®^ Bead Tubes Type B (Macherey Nagel, Düren, Germany) and a tissue lyser (Quiagen, Germany) (5 times, 1 min., 60 Hz). After each step, the samples were cooled on ice.

The isolation and purification of RNA from tracheal cells was performed according to the manufacturer’s instructions using the NucleoSpin RNA II Kit (Macherey Nagel, Düren, Germany). The total RNA was eluted in 40 µl sterile H_2_O (RNAse-free). Afterwards an additional digestion of the remaining DNA was performed using the turbo DNAse free kit (Thermo Fisher Scientific, Waltham, USA). Therefore 10 µl Turbo DNase buffer (10x), 1 µl Turbo DNase and 5 µL RNase-free water were added to each batch. The reaction mix was incubated at 37 °C for 30 min. Then 5 µl inactivation buffer was added, incubated for 5 min at room temperature followed by a centrifugation step (10,000 x g; 1.5 min), 40 µl supernatant were transferred in a new tube and the concentration and purity of the RNA was determined using a nanophotometer (P330, Implen, Germany).

For synthesis of cDNA, Hexamere random primes (ThemoFisher Scientific, Germany) and SuperScript^®^ III reverse transcriptase according to the manufacturer’s protocol were used. For all tracheal samples, 100 ng of RNA were used in a total reaction volume of 20 µl. For all negative control samples, the maximum RNA volume of 11 µl was used.

#### Library preparation and sequencing

We selected the primers S-D-Bact-0008-a-S-16 (5’-AGAGTTTGATCMTGGC-3’) and S-D-Bact-0343-a-A-15(5’-CTGCTGCCTYCCGTA-3´), which amplifies the hypervariable regions V1-2 of the 16 S rRNA gene [11] with added overhanging sequences at their 5′ ends compatible to Nextera XT indices for multiplexing. PCR reactions contained 50 ng template cDNA. PCRs were performed in tripliactes and negative template PCR controls were included in each run. PCR conditions were as follows: 10 s of initial denaturation at 98 °C; 22 cycles including denaturation at 98 °C for 30 s, 45 s annealing at 58 °C and 30 s elongation at 72 °C; a final 5 min elongation step at 72 °C. Success of each PCR was verified by agarose gel electrophoresis. PCR products were purified with Agencourt AMPure XP paramagnetic beads (Beckman Coulter, Brea, USA) and then checked for dimers and were quantified using the DNF-473 Standard Sensitivity NGS Fragment Analysis Kit on the Fragment Analyzer (Advanced Analytical, Ankeny, USA). Library preparation was performed according to the Illumina guidelines for 16 S rDNA gene amplicon preparation with slight modifications [12] with 8 cycles of indexing PCR. Following purification and quality control of the indexing, PCR products were diluted to 4 nmol/l and pooled. Sequencing was performed on a MiSeq^®^ System (Illumina, Inc., CA, USA) using the MiSeq^®^ Reagent Kit v3 (600 cycles) for paired end sequencing according to the manufacturer’s guidelines. Sequences are deposited at NCBI (SRA number SUB10670274, bioproject PRJNA784260).

#### Sequence processing

Raw reads were processed according to the FASPA protocol [13]. In short, demultiplexed raw reads were quality filtered and merged using USEARCH v.10.2.240 [14]. After removal of sequences below a maximum expected error threshold of 1.0, primer stripping and sequence trimming in USEARCH, we applied the Unoise3 algorithm to identify zero-radius operational taxonomic units (zOTUs) [15]. Only zOTUs with a minimum length of 270 bp were kept and the resulting zOTU table was reanalyzed using the UNCROSS algorithm [16] to remove sequencing errors due to erroneous assignment of barcodes. We applied the SINTAX algorithm with a confidence cut-off at 0.5 [17] on the RDP reference database v16 [18] to assign the taxonomy on zOTUs down to the genus level. Estimation of species level was performed for selected zOTUs using blastn on the nt database. Assignments on the species level were made in case the zOTU had an unambiguous 100 % similarity to an isolate/cultivate of which the full 16 S rRNA gene sequence was available. We applied the cluster–agg command in USEARCH v.10.2.240 to construct a phylogenetic tree in Newick format. The final zOTU table was analyzed for contaminations.

PCR negative controls had few reads belonging to distinct bacterial lineages and were removed from further analysis. zOTUs of above-described negative control samples were compared to the zOTUs of the respective larvae/tracheae samples to estimate the impact of contamination introduced at each step. Thus, contaminated larvae/tracheae samples were removed at this step.

### Statistical analyses

A FASPA script [13] was applied to enable downstream analyses in R [19]. Using the *Rhea* pipeline [20] we performed rarefaction analysis to control for the sufficiency of sequencing depth. Following sample normalization, *Rhea* scripts were also used for the estimation of α – and ß-diversity, serial group comparisons and correlation analyses. α-Diversity measures included observed species richness, Shannon diversity and Evenness; For ß-diversity Nonmetric multidimensional scaling (nMDS) analyses were calculated based on generalized UNIFRAC distances [21]. Differences of categorical metadata among all samples analyzed were calculated using PERMANOVA (999 permutations). On the taxa and zOTU levels, pairwise categorical differences were calculated using the Wilcoxon-Rank-Sum-Test with FDH correction. For Pearson correlation (FDH corrected) analysis, the quantitative relative abundance data of zOTUs and taxa was center log-transformed to remove compositional constraints.

## Results

**Fig. 1:**
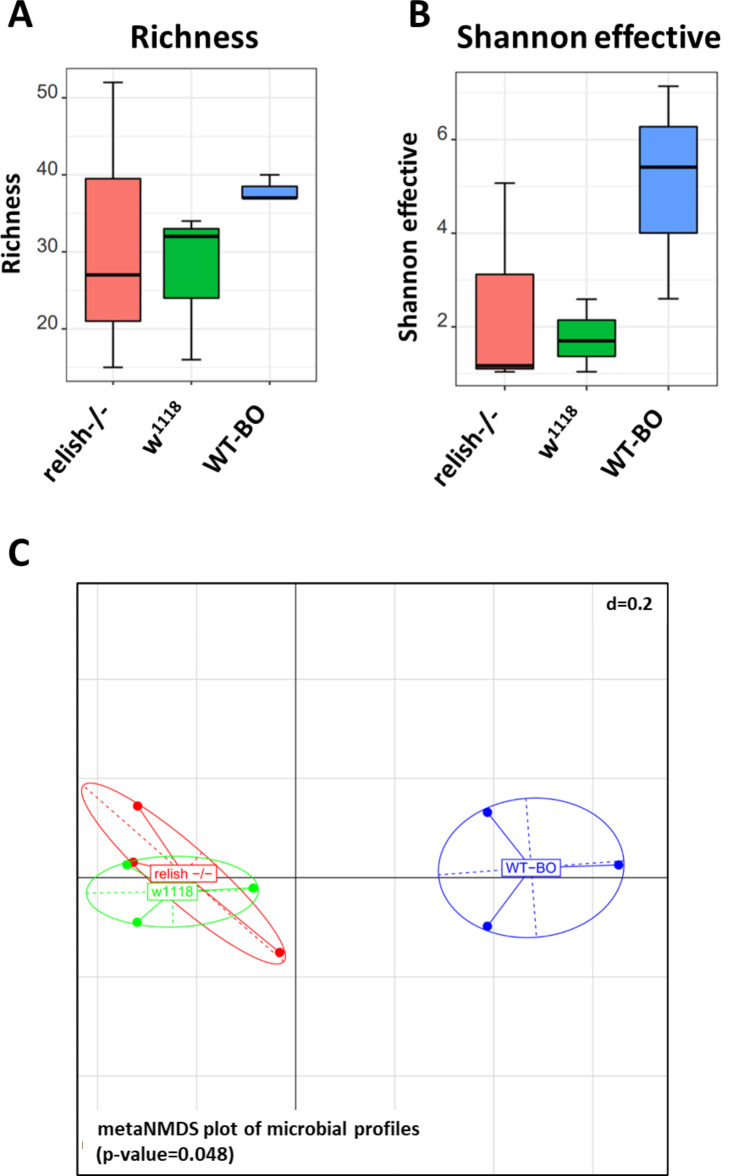
α-diversity zOTU Richness **(A)**, Shannon effective **(B)** and unconstrained nonmetric multidimensional scaling (metaMDS) plot **(C)** in dissected tracheae in different fly strains **A**: α-diversity metrics based on zOTU richness and **B**: Shannon diversity in dissected tracheae in w^1118^ (red), relish^−/−^ (green) and WT-BO (blue). Wilcoxon-Rank-Sum test, corrected for multiple testing with the Benjamin-Hochberg method to decrease the False Discovery Rate (FDR). N = 3 per group. **C**: Unconstrained nonmetric multidimensional scaling (metaMDS) plot of generalized UniFrac distances. w^1118^(green), relish^−/−^ (red), WT-BO (blue), p < 0.048 (ANOSIM). N = 3 per group

**Fig. 2:**
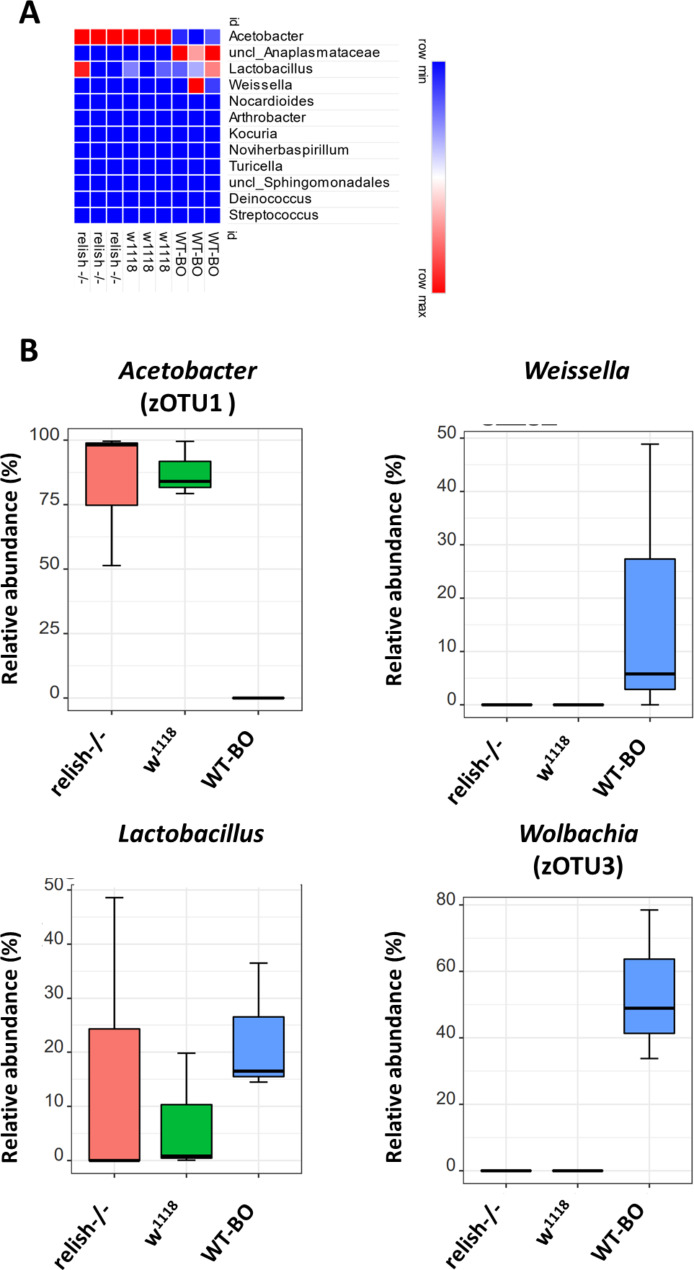
Abundance of bacterial families **(A)** and influence of strain type on bacterial taxa and zOTUs **(B)** in isolated airways of different *Drosophila* strains **A**: Relative abundance of bacterial families in dissected larval tracheae are displayed. Each bar represents individual *D. melanogaster* samples. The different *D. melanogaster* strains are indicated at the bottom of the bar charts. **B**: Box plots showing significantly different median relative abundances of genera and zOTUs between groups. Pairwise Wilcoxon Rank Sum test (p < 0.05, FDR corrected). N = 3 per group

On average we obtained 68,574 high quality reads per sample, which was sufficient to cover the bacterial diversity in tracheae from larvae of the different *Drosophila* specimen. We observed a relatively high α-diversity of bacteria in tracheae of larvae which derived from wild-caught specimen as compared to lab strains (Fig. 1A, B). Estimation of ß-diversity indicated that the microbial community composition of larvae from wild-caught WT-BO is distinct from the lab strains w^1118^ and relish^−/−^ (Fig. 1C; ANOSIM with 999 perturbations; p = 0.048). The phylogenetic analysis revealed a high degree of similarity in the larvae, which originated from the lab-strains, which were both dominated by the two families *Acetobacteraceae*, and *Lactobacillaceae*, while larvae from wild-caught specimens were dominated by *Lactobacillaceae, Anaplasmataceae* and *Leuconostocaceae* (Fig. 2A).

Most reads allocated to *Acetobacteraceae* obtained from larvae of the laboratory strains were assigned to zOTU1 which accounted for > 80% of reads in tracheae but was absent in WT-BO (Fig. 2B; Table 1). *Lactobacillaceae* represented six of the eight most abundant zOTUs (Table 1) and were equally present in all *D. melanogaster* specimens.

**Table 1:** Abundant and prevalent bacterial zOTUs in *D. melanogaster* larvae. The zOTUs listed contribute to at least 1% of total reads across all samples taken of each *D. melanogaster* strains. To obtain the nearest named isolate, the representative sequence for each zOTU was compared to the NCBI database for nucleotide blast (status 24.02.2021)

WT-BO			
zOTU ID	% of total reads	% of samples	Nearest named isolates (identity)
**zOtu3**	57,72%	100%	*Wolbachia pipientis*
**zOtu13**	10,49%	66,67%	*Weissella hellenica*
**zOtu16**	8,55%	66,67%	uncultured bacterium
**zOtu35**	8,28%	66,67%	*Acetobacter sicerae*
**zOtu5**	6,96%	100,00%	*Lactiplantibacillus plantarum*
**zOtu29**	5,65%	66,67%	*Lactiplantibacillus plantarum*
**zOtu7**	5,29%	100%	*Lactiplantibacillus plantarum*
**zOtu26**	4,55%	66,67%	*Weissella hellenica*
**zOtu34**	3,74%	66,67%	*Weissella hellenica*
**zOtu11**	3,51%	66,67%	*Lactiplantibacillus plantarum*
**zOtu51**	3,21%	66,67%	*Acetobacter sp.*
**zOtu6**	2,83%	66,67%	*Lactiplantibacillus plantarum*
**zOtu9**	2,07%	66,67%	*Lactiplantibacillus plantarum*
**w** ^**1118**^			
**zOTU ID**	**% of total reads**	**% of samples**	**Nearest named isolates (identity)**
**zOtu1**	87,61%	100%	*Acetobacter pomorum*
**zOtu4**	9,51%	66,67%	*Levilactobacillus brevis*
**relish** ^**−/−**^			
**zOTU ID**	**% of total reads**	**% of samples**	**Nearest named isolates (identity)**
**zOtu1**	83,89%	100%	*Acetobacter pomorum*
**zOtu6**	3,22%	33,33%	*Lactiplantibacillus plantarum*
**zOtu5**	3,18%	33,33%	*Lactiplantibacillus plantarum*
**zOtu7**	2,66%	33,33%	*Lactiplantibacillus plantarum*
**zOtu9**	2,50%	33,33%	*Lactiplantibacillus plantarum*
**zOtu4**	1,89%	33,33%	*Levilactobacillus brevis*

The high abundance of *Anaplasmataceae* in airways of larvae from wild-caught specimen was assigned to *Wolbachia sp.* (zOTU3), one of the most widespread maternally transmitted, intracellular pathogen in arthropods [22]. *Wolbachia sp.* is known from other studied on the role of the microbiome for insects to influence the behavior and development in *D. melanogaster* [23]. The second most abundant family in two out of three samples of larvae from wild-caught flies was *Leuconostoceae*, with the most abundant zOTU being assigned to *Weissella sp.* (Fig. 2B; Table 1).

Finally, a relative high abundance of *Serratia* sp. (zOTU36) was found in larvae from relish^−/−^ compared to w^1118^ and WT-BO.

## Discussion

In the present study, we demonstrate for the first time that *D. melanogaster larvae* harbor an airway microbiome that is of low complexity. As described in more detail in the methods section, contamination of our samples by environmental bacteria is unlikely as tracheal preparation was performed under highly sterile conditions using only gas-filled, i.e. undamaged tracheae. Together with the use of non-template extraction controls and a rigorous elimination of the very few zOTUs found in the negative control, for downstream analysis (see supplemental material), we are confident that we did not include any contaminating reads into our analysis. Similarly, contamination with gut bacteria can be ruled out since the intestine is not damaged during tracheal isolation (see also video in supplemental methods section). Along this line a study by Fink et al. [24] compared data of whole fly, midgut and faeces of three different fly lab strains, including w^1118^, which was also used in our study. The authors found similar taxa in all three sample types, indicating an overlap of species found in gut and whole *D. melanogaster*. However, specific qPCR analysis enabled PCoA clustering of *A. tropicalis*, *Commensalibacter intestini*, *L. brevis*, *L. plantarum*, and *Guconobacter sp*. according to organs. Of note, for w^1118^ there was a very low abundance of *Gluconobacter sp.* and *C. intestini* in whole flies compared to the gut and fecal samples, indicating very low levels in organs outside the gut. We therefore used these species as indicators of potential gut contaminations but found only very few numbers of zOTUs in all samples of our fly strains. We therefore provide here another line of evidence that no contaminating gut DNA was co-extracted in our samples.

The lower α-diversity of laboratory strains w^1118^ and the immune deficient line relish^−/−^ compared to larvae which were obtained from flies caught in nature maybe explained by the limited contact of the flies with environmental microbes under uniform culture conditions on semi-sterile medium [25–27]. As eggs from laboratory and wild caught flies were laid and cultivated under identical conditions, differences in the airway microbiome must be linked to vertical microbial transmission from the parental generation via eggs to the offspring larvae.

*Lactobacillaceae and Acetobacteraceae* prevailed in the tracheal system of all larvae independent of the origin of the parental flies. Both taxa have been shown to be prevalent in gut microbial communities of *D. melanogaster* laboratory strains earlier [26, 28] and were considered important for successful oogenesis [29], development and survival [30]. The presence of those families also in the tracheal system of the larvae indicates that at least at younger development stages the different organs of the flies might be provide comparable habitats for microbial colonization. However, it cannot be excluded that differences in the ecophysiology of the microbes colonizing gut and tracheal system might be present and only visible on a higher taxonomic level (species or strains).

The higher relative abundance of *Serratia marcescens* in larvae of the immunodeficient laboratory strain is in line with the already described susceptibility to intestinal *Serratia marcescens* infections in immunosuppressed *D. melanogaster* strains [31].

## Conclusions

In summary, we demonstrate for the first time that larvae of *D. melanogaster* harbors an airway microbiome. We further show that also in airways, the microbial composition clearly differs between laboratory and wild-caught strains and is mainly shaped by environmental conditions and at least partly transmitted to the next generation. However, our study also provides evidence that the conditions under which laboratory strains are commonly kept can lead to a significant loss of diversity in the host-associated microbiome over many generations. Smaller differences between the wildtype and immunodeficient lab strains on genus level indicate an additional influence of the immune status on the microbial composition. Further studies are needed to understand the impact of the airway microbiome on the fly´s resilience towards airborne biotic or abiotic stressors.

## Electronic supplementary material

Below is the link to the electronic supplementary material.

Supplementary Material 1

Supplementary Material 2

## Data Availability

The datasets generated and/or analysed during the current study are available in the Sequences are deposited at NCBI (SRA number SUB10670274, bioproject PRJNA784260) repository, [https://www.ncbi.nlm.nih.gov/bioproject/PRJNA784260/]. All data generated or analysed during this study are included in this published article [and its supplementary information files]. Sequences are deposited at NCBI (SRA number SUB10670274, bioproject PRJNA784260).
