# Supplementary material for: The microbial composition of larval airways from Drosophila melanogaster differ between specimens from laboratory and natural habitats
Source: Environ Microbiome. 2023 Jun 27;18:55. doi: 10.1186/s40793-023-00506-9 (PMC10303296; doi:10.1186/s40793-023-00506-9)
Supplement: Supplementary file 1 — Supplementary Material 1 [file 40793_2023_506_MOESM1_ESM.pdf]

Supplemental Material

Table S1: *D. melanogaster* strains used for analysing the larval airway microbiome.

Sample no.	Fly strain	Genotype	Collection Date	Dissection Date	Origin
**1**	WT-BO	N/A	25.06.2018	10.07.2018	Domestic kitchen, Bad Oldesloe (Germany)
**2**	WT-BO	N/A	28.06.2018	10.07.2018	Domestic kitchen, Bad Oldesloe (Germany)
**3**	WT-BO	N/A	01.07.2018	10.07.2018	Domestic kitchen, Bad Oldesloe (Germany)
**4**	w^{1118}	w[1118]	04.07.2018	10.07.2018	Thomas Roeder, Zoological Institute, University of Kiel
**5**	w^{1118}	w[1118]	23.04.2019	29.04.2019	Thomas Roeder, Zoological Institute, University of Kiel
**6**	w^{1118}	w[1118]	23.04.2019	29.04.2019	Thomas Roeder, Zoological Institute, University of Kiel
**7**	relish-/-	w[1118]; Rel[E38] e[s]	04.07.2018	10.07.2018	Bloomington Drosophila Stock Center, Indiana University, Bloomington (USA); RRID:BDSC_9458
**8**	relish-/-	w[1118]; Rel[E38] e[s]	24.04.2019	30.04.2019	Bloomington Drosophila Stock Center, Indiana University, Bloomington (USA); RRID:BDSC_9458
**9**	relish-/-	w[1118]; Rel[E38] e[s]	24.04.2019	30.04.2019	Bloomington Drosophila Stock Center, Indiana University, Bloomington (USA); RRID:BDSC_9458
